# Number comparison under the Ebbinghaus illusion

**DOI:** 10.3389/fpsyg.2022.989680

**Published:** 2022-09-20

**Authors:** Wei Liu, Chunhui Wang, Xiaoke Zhao, Shixin Deng, Yajun Zhao, Zhijun Zhang

**Affiliations:** ^1^School of Education, Yunnan Minzu University, Kunming, China; ^2^School of Education, Dali University, Dali, China; ^3^School of Sociology and Psychology, Southwest University for Nationalities, Chengdu, China; ^4^Department of Psychology and Behavioral Sciences, Zhejiang University, Hangzhou, China

**Keywords:** numerosity perception, density perception, the Ebbinghaus illusion, perceived area, Weber fraction

## Abstract

A series of studies show interest in how visual attributes affect the estimate of object numbers in a scene. In comparison tasks, it is suggested that larger patches are perceived as more numerous. However, the inequality of density, which changes inversely with the area when numerosity remains constant, may mediate the influence of area on numerosity perception. This study aims to explore the role of area and density in the judgment of numerosity. The Ebbinghaus illusion paradigm was adopted to induce differences in the perceived, rather than the physical, area of the two patches to be compared. Participants were asked to compare the area, density, and the number of the two patches in three tasks. To this end, no PSE (point of subjective equality) bias was found in number comparison with randomly distributed dots, although a significant difference was revealed in the perceived area of the two patches. No PSE bias was found in the density comparison, either. For a comparison, density and number tasks were also conducted with regularly distributed dots. No PSE bias was found in density comparison. By contrast, significant PSE bias showed up in number comparison, and larger patches appeared to be more numerous than smaller patches. The density mechanism was proposed as the basis for number comparison with regular patterns. The individual Weber fractions for regular patterns were not correlated with those for random patterns in the number task, but they were correlated with those for both patterns in the density task. To summarize, numerosity is directly sensed, and numerosity perception is not affected by area inequality induced by the Ebbinghaus illusion. In contrast, density and area are combined to infer numerosity when the approximate numerosity mechanism is disrupted by dot distribution.

## Introduction

Evidence suggests we have the ability to perceive the number of a scene at a glance, which is revealed both in studies of adults and newborns, as well as in studies of nonhuman species such as bees and fish (Dehaene, 2002; Leibovich et al., 2017). Studies showing that numerosity perception is independent of texture analysis highlight the signature of a direct number sense (Dehaene and Changeux, 1993; Burr and Ross, 2008). Perceiving of numerosity is demonstrated to be more sensitive than density or area when stimuli features vary along the three dimensions (Cicchini et al., 2016, 2019).

There are, however, studies that contend numerosity perception is a by-product of texture analysis, for example, observers may infer the number of items by combining the density and spatial extent of stimuli. It is revealed that perceived numerosity is biased according to the distributing area. When patches to be compared are unmatched in sizes, participants make errors consistent with larger patches being more numerous and denser (Dakin et al., 2011; Gebuis and Reynvoet, 2013; Beran and Parrish, 2016; Yousif and Keil, 2020). Although number estimates can be slightly influenced by area or density, there is also evidence that number has a stronger influence on density and area, suggesting that number is the more basic attribute (Cicchini et al., 2016; Burr, 2017). Nevertheless, the fact that systems interact with each other does not preclude the existence of a dedicated number mechanism (Burr, 2017).

In most studies, unmatched patches can produce area and density inequality simultaneously when the item number is held constant (Dakin et al., 2011; Gebuis and Reynvoet, 2013; Beran and Parrish, 2016; Yousif and Keil, 2020). Due to the inequality in density between unmatched patches, the effects of the area on number perception can be confusing. Numerosity perception could be modulated by unmatched patch area, or by the change in density which is induced by varying areas. Furthermore, the effect can also be explained by the fact that distinct mechanisms are activated in number compared with unmatched patches. For example, in the study of Dakin and colleagues (2011), when the two patches to be compared were equal in the item number, dots in the smaller patch were evidently too dense to be separated, preventing the activation of the approximate numerosity mechanism (Anobile et al., 2014, 2015). In this case, number comparison can be mediated by density rather than numerosity. As a result, the special task paradigm may also be responsible for the effects.

In this study, we explored the role of area and density in the judgment of numerosity. To isolate the perceived area, density, and numerosity, the Ebbinghaus illusion (Titchener, 1905) was adopted to induce the difference in a perceived area of two patches presenting stimulus dots, whereas the physical area of these patches was kept constant. In a previous study, size adaptation was used to manipulate the apparent size of the numerosity patch while maintaining its physical size and density. As compared with high numerosities (50–100), low numerosities (4–25) were much less affected by changes in apparent size, and density perception was invariant to changes in apparent patch size for all numerosities (Zimmermann and Fink, 2016). In the current study, we hypothesized that the perception of density would remain unaffected by apparent size changes. Further, for randomly distributed patterns with a moderate number of dots, which were proposed to activate the numerosity mechanism (Anobile et al., 2014), varying the area was not expected to have a significant effect on the perception of numerosity.

To make a clearer comparison, regular distribution patterns in which dots are distributed in metrics were also adopted. According to a series of studies, number comparison with regular patterns may engage the density mechanism (Liu et al., 2017, 2018, 2022). First, the effect of connectedness is much weaker for regular patterns, suggesting that individuation is inhibited, possibly because the highly regular patterns emphasize the structure of the “whole” (Liu et al., 2017). Second, changes in stimulus orientation and size have no effect on adaptation for random patterns, but significantly affect numerosity adaptation in regular patterns (Liu et al., 2017, 2018). Third, the adaptation of regular patterns is monocular transferring (adaptation aftereffects remain in the exposed eye), whereas the adaptation of random adaptors is binocular transferring (adaptation aftereffects can transfer to the unexposed eye (Liu et al., 2017). Fourth, in number comparison tasks, P2p amplitudes over right occipital-parietal sites are found to be weaker for regular patterns than for random patterns, which is consistent with the ERP differences between processing mechanisms based on density and numerosity (Fornaciai and Park, 2017; Liu et al., 2022). ERP and behavioral dissociation suggest that regular distribution can trigger density processing in numerosity comparison tasks. As dense patterns also possess high distribution regularity, it is not unreasonable to hypothesize that high density can serve as a special case of high regularity in activating the density mechanism.

In the current study, we went on to investigate number processing with regular and random patterns. By comparing the two patterns with identical testing dot number potential variables can be excluded. We hypothesized that the judgement in the density task with regular patterns will not be biased by changes in the area, whereas the effect of unmatched patch area on number comparison with regular patterns is much stronger than that with random patterns. The error in number task would be consistent with that the patch perceived as larger also appears to be more numerous. According to our hypotheses, changes in the perceived area will not affect the perception of density, and number comparison with regular patterns is mediated by density and area analyses. For regular patterns, if numerosity is inferred on the basis of area and density, the larger patch will be judged as more numerous, because the larger one should contain more dots when the two patches are equally dense.

In summary, this study examined the role of area and density in number comparison when patches are unmatched in the perceived area. The Ebbinghaus illusion was adopted to induce differences in perceived area, and density and number tasks were investigated with stimuli distributed in two patterns. To anticipate the results we found that for regular patterns, inequality in the perceived area does not affect PSE in the density task, whereas it results in a significant overestimation of PSE for dots in the larger patch in the number task. Individual Weber fractions (Anobile et al., 2014) of comparison with random and regular patterns are not correlated with each other in number task, whereas the Weber fractions of regular patterns in number task are significantly correlated with those of both patterns in the density task, providing further evidence supporting that the mechanism of density, rather than numerosity, is activated by regular patterns even when participants are asked to compare the number. When the approximate numerosity mechanism is disrupted by dot distribution, numerosity is inferred by combining density and area, and unequal area influences numerosity perception. For random patterns, on the contrary, PSE is not biased by unmatched patch area, either in density or number tasks. No correlation is revealed for Weber fractions between number and density tasks. The results suggest that numerosity perception is independent of density perception and that numerosity is sensed directly.

## Materials and methods

### Statement

All administered measures and tested experimental conditions were reported for all experiments. The calculation of individual Weber fractions excluded Weber fractions beyond three standard deviations of the mean. For participants with deleted data, individual Weber fractions were calculated by averaging their rest data under equivalent conditions. As a result, data of all participants were reserved for calculation. Out of 544 original Weber fractions, nine were excluded (1.6%).

### Ethics statement

For all experiments, the data were analyzed anonymously. Participants provided their informed consent verbally and in writing. The Ethics Committee of Yunnan Minzu University approved this study.

### Participants

With an α error probability of 0.05 and a power of 0.8, power analysis using G*power 3.1 showed that at least eighteen participants were required for repeated measures ANOVA with a 0.4 effect size, and at least 32 participants were required for correlation analyses with a 0.3 effect size. A total of 34 adults (age range = 19–33 years; 19 males) participated in the main tasks. In addition, another 8 adults (20–29 years; 3 males) participated in the control experiment to estimate the magnitude of the illusion. All adults in this study were right-handed with normal or corrected-to-normal vision.

### Apparatus

The stimuli were presented on a 19” flat-screen monitor with a resolution of 1,600 × 900 pixels and a refresh rate of 60 Hz in a dark room. The monitor was located approximately at 45 cm from the seated participant. All the stimuli were displayed using E-prime 1.0 (PST, Sharpsburg, PA, United States) software.

### Stimuli

**Figure 1:**
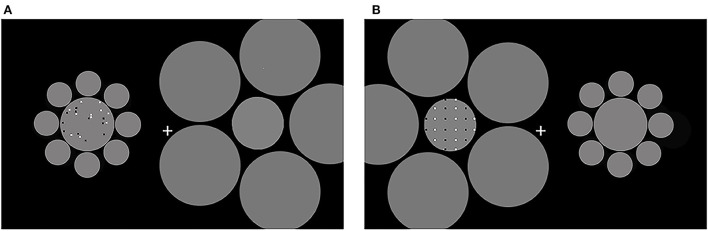
The stimuli used in the current study. Reference in the random and the regular conditions are shown. Reference and test patterns were displayed sequentially, each in one patch. To induce the Ebbinghaus area illusion, the patches were surrounded by larger and smaller circles, respectively. **(A)** A sample for random patterns; **(B)** a sample for regular patterns. The positions of references and tests were counterbalanced across participants.

Dot-array stimuli were displayed within two fixed circular patches centered at 13° from the middle of the computer screen (Figure 1). Each gray-scale pattern (RGB: 128, 128, 128) had a diameter of 8.9° (250 pixels) and was presented against a black (RGB: 256, 256, 256) background. To induce the Ebbinghaus illusion, one patch was surrounded by five larger circles (RGB: 128, 128, 128) with a diameter of 12.6° (450 pixels), and the other patch was surrounded by eight smaller circles with a diameter of 2.8° (100 pixels).

Participants were asked to compare the reference with a series of tests, using the Method of Constant Stimuli. On each trial, two sets of dots were presented in succession in the patches, one as a reference and one as a test. There were 40 square dots in each reference (20 white and 20 black) with a diameter of 0.25°. Eight tests contained 24, 30, 33, 36, 44, 49, 58, or 68 dots, respectively. A log scale was used to determine the number of tests. Besides, we chose the number with which symmetric patterns can be constructed with regular patterns. In random patterns, dots were pseudo-randomly distributed in reference and test patterns with the restriction that there was no overlap for dots (Figure 1A). In regular patterns, dots were regularly distributed. Black and white dots were arranged into vertical lines. Black and white lines were composed of dots that were presented in turn from left to right (Figure 1B).

According to the study by Anobile et al. (2015), the switching point from numerosity to density mechanism is centered at 40 dots (0.8 dots/^°2^, for reference) at 15° eccentricity with test series. In this study, stimuli were presented at 13° from the center. The reference contained 40 dots (0.6 dots/^°2^). According to these parameters, the stimulus used is in the numerosity regime.

### Procedure

The testing stage used the point of subjective equality (PSE) and the just noticeable difference (JND) to assess numerosity perception. Weber fraction was estimated by the division between JND and PSE (Anobile et al., 2014). A 2AFC (temporal) task was adopted to produce a psychometric function (dependent variable: the probability of test > reference) from which the PSE and JND were extracted as measures of perception. Two sets of dots were shown sequentially in the two fixed circles in the horizontal direction, and each trial was presented to participants with forced-choice questions: “Which circle is denser?” in the density task, and “Which circle contained more dots?” in the number task. To respond, participants pressed buttons: “f” with their left hand denoted that the left circle was denser/more numerous, whereas “j” with their right hand indicated that the right circle was denser/more numerous.

**Figure 2:**
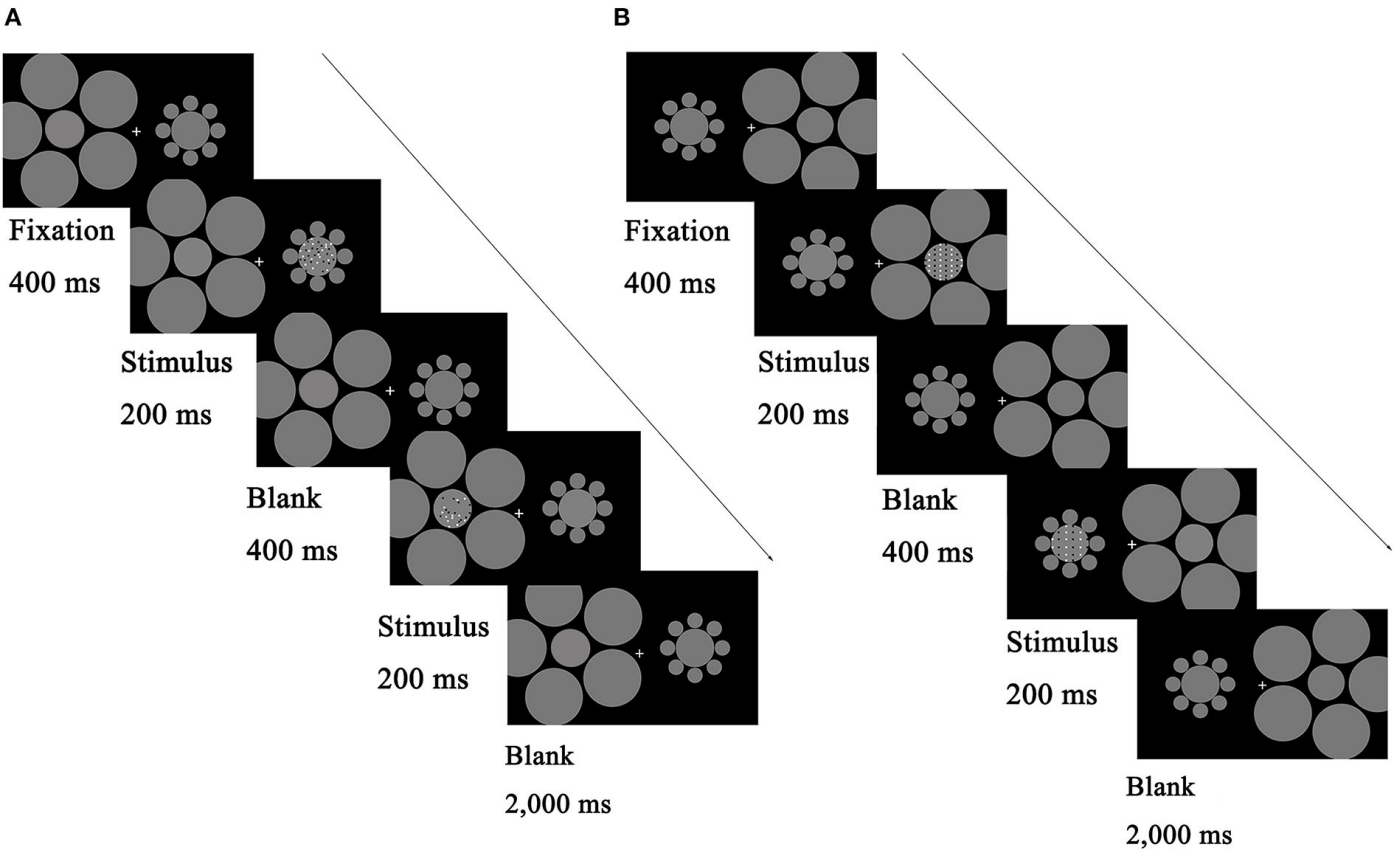
Schematic illustration describing the procedure of the main tasks. Each trial began with a fixation for 400 ms, and then the test stage began with test stimuli displayed on one side of the screen for 200 ms, followed by reference stimuli on the other side for 200 ms. The two stimuli were separated by a blank screen lasting 400 ms. Participants reported which patterns (left or right) appeared to be denser/more numerous; they guessed when they were unsure. **(A)** Comparison task with random patterns. **(B)** Comparison task with regular patterns. The test and reference positions (left or right) and the presented patches (perceived to be smaller or larger) were counterbalanced across participants.

The procedure is described in Figure 2 (A: random condition; B: regular condition). Participants initiated the experiment by pressing the space bar. A fixation point was shown in the center of the screen for 400 ms, followed by the left circle with the test stimuli for 200 ms. Then, the right circle with a reference stimulus was shown for 200 ms. A blank frame with a fixation cross isolated the test and the reference for 400 ms. The next trial began either after the participants responded or after 2,000 ms. The test and reference positions (left or right) and the presented patches (perceived to be smaller or larger) were counterbalanced across participants.

To improve participants' familiarity with the experiment, a 2-min practice session with feedback was conducted at the start of the experiment. Then, participants completed the formal experiment. This study adopted a 2 (task: density/number) × 2 (dot patterns: random/regular) × 2 (perceived patch area: larger/smaller) × 2 (reference location: left/right) within-subject design. Hence there are 16 levels for each participant. Each test was compared to the reference for eight trials at each level, for a total of 64 trials. Overall, the 1,024 trials were divided into 16 blocks with counterbalanced sequences for each participant, with adequate rest between blocks to prevent fatigue.

To test the bias in area perception induced by the Ebbinghaus illusion, an size task was also conducted. As a reference, we used a patch with a diameter of 250 pixels, which was identical to the patches used in the density and number tasks. This reference was surrounded by smaller circles of 100 pixels. Eight tests with varied diameters of 200, 215, 230, 240, 265, 288, 313, and 338 pixels were surrounded by larger circles of 450 pixels. Participants were asked to compare which of the two patches was larger. Each illusion test contained 64 trials, and there were two illusion tests in which the reference appeared on the left and right sides respectively. At baseline, participants also completed two 64-trial tasks with identical references and tests, but those patches were not surrounded by any circles. To determine whether the Ebbinghaus illusion caused overestimation in the perceived area for reference, 256 trials were conducted.

### Statistical analysis

Individual PSE, JND, and Weber fractions were calculated to estimate the accuracy and precision of comparison. Paired *t*-tests were two-tailed. Pearson correlation coefficient *r* was calculated for Weber fractions between two conditions. Cohen's *d* was reported to provide a complement to null hypothesis statistical significance testing by estimating the magnitude of the difference (0.2–0.5 for small effect size, 0.5–0.8 for moderate effect size, and >0.8 for large effect size). Bayes factors (*BF*_10_) were reported to estimate whether the null hypothesis H_0_ or the alternative hypothesis H_1_ is more likely to be correct. *BF*_10_ < 0.3 suggests clear evidence for H_0_, whereas *BF*_10_ > 3 indicates clear evidence for H_1_. (Bayes) repeated-measures ANOVA was conducted, and $\eta_{p}^{2}$ was reported to estimate the effect of independent variables. The False Discovery Rate probability (FDR) was adopted to correct the probability of type-I error in multiple comparisons with *Q* probability (Liu et al., 2020). For example, with four *p* values in multiple comparisons, multiply the smallest *p*-value by four to get its *Q* value. Then multiply the second smallest *p* by 4/2, the third *p* by 4/3, and the last *p* (the largest one) by 4/4 to get their *Q* values. To determine whether a comparison is statistically significant, *Q* values are used instead of *p* values, ^*^Q < 0.05, ^**^Q < 0.01, ^***^Q < 0.001. Figures were generated in Matlab (MathWorks, Natick, MA, United States) and Origin 9 (OriginLab, Northampton, MA, United States).

## Results

### Size task

**Figure 3:**
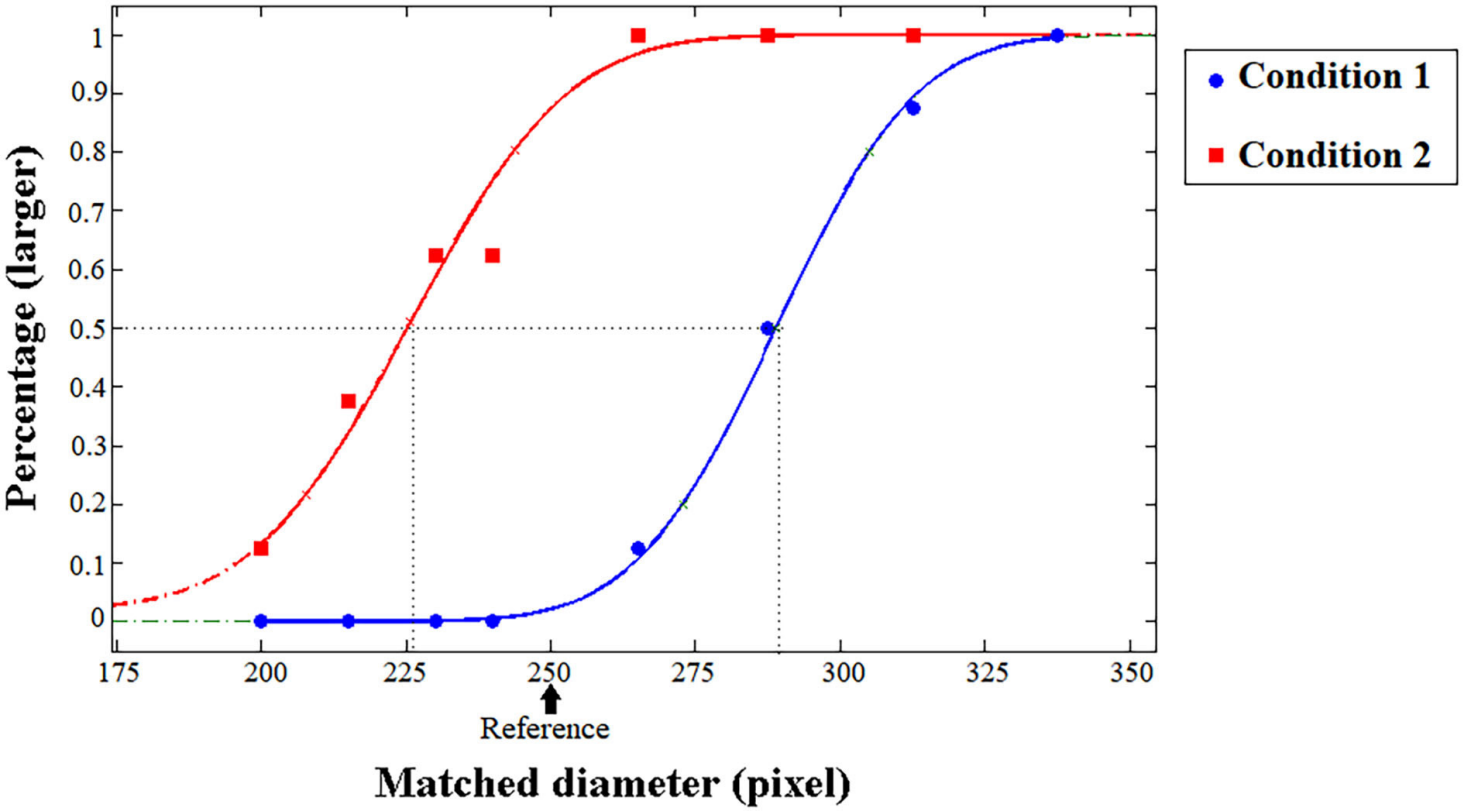
Typical psychometric functions in the size task (control experiment). The proportion of trials in which the test patch appeared to be larger than the reference patch was plotted as a function of the pixel of the test patch, and the vertical dashed lines indicate the PSE. The arrow shows the reference diameter (pixel). The participants' typical responding curves are displayed to indicate the average PSE results. The blue line represents Condition 1 (the reference was surrounded by smaller circles), while the red line represents Condition 2 (the reference was surrounded by larger circles).

Using the psignifit toolbox version. 2.5.41 of MATLAB (MathWorks, Natick, MA, United States; Figure 3), a cumulative Gaussian function was fitted to the proportion of participants' responses to measure the accuracy and precision of participants' perception. The values of the test stimuli (abscissa) corresponding to the 50% points were calculated from the fit curves. These values are the PSE representing the area of test patches that appeared equal to the area of reference patches, according to the participants. For the illusion test, PSE = 286.26 (SD = 16.59), 95% CI = [280.47, 292.05]. For baseline, PSE = 257.57 (SD = 5.21), 95% CI = [255.75, 259.39]. Significant overestimation was found in illusion condition compared with baseline, *t*(33) = 9.128, *p* < 0.001, Cohen's *d* = 1.565, *BF*_10_ > 100. Unequal perceived sizes for the patches were induced by the Ebbinghaus illusion.

To minimize differences with the density and number task, a control experiment was conducted with dots presented in the center circles. A total of 16 participants (half of whom also participated in the main tasks) were asked to compare the sizes of reference and test patches. In Condition 1, the reference (250 pixels) was surrounded by smaller (100 pixels) circles, while the tests were surrounded by larger (450 pixels) ones. In Condition 2, the surrounding circles were inverse. In total, 24–68 dots were randomly spread out in the center patches, and in different blocks, dots were distributed randomly or regularly. Figure 3 shows typical psychometric functions in the control experiment. For Condition 1, PSE = 289.82 (SD = 10.46), 95% CI = [284.25, 295.39] for random patterns, and PSE = 290.76 (SD = 7.13), 95% CI = [286.96, 294.57] for regular patterns. For Condition 2, PSE = 227.68 (SD = 17.42), 95% CI = [218.40, 236.96] for random patterns, and PSE = 234.30 (SD = 15.12), 95% CI = [226.25, 242.36] for regular patterns. Significant overestimation was found between conditions for random patterns, *t*(15) = 9.831, *p* < 0.001, *Cohen's d* = 2.458, *BF*_10_ > 100, and for regular patterns, *t*(15) = 12.831, *p* < 0.001, *Cohen's d* = 3.208, *BF*_10_ > 100. The illusion magnitude is defined as the diameter difference between the center patches surrounded by small and large inducers, compared to the standard patch size (Takao et al., 2021). The overall illusion magnitude is 25% for random patterns (2.2°), and 23% for regular patterns(2.0°), which is in accordance with the related studies (Chen et al., 2021; Takao et al., 2021).

### Density task

**Figure 4:**
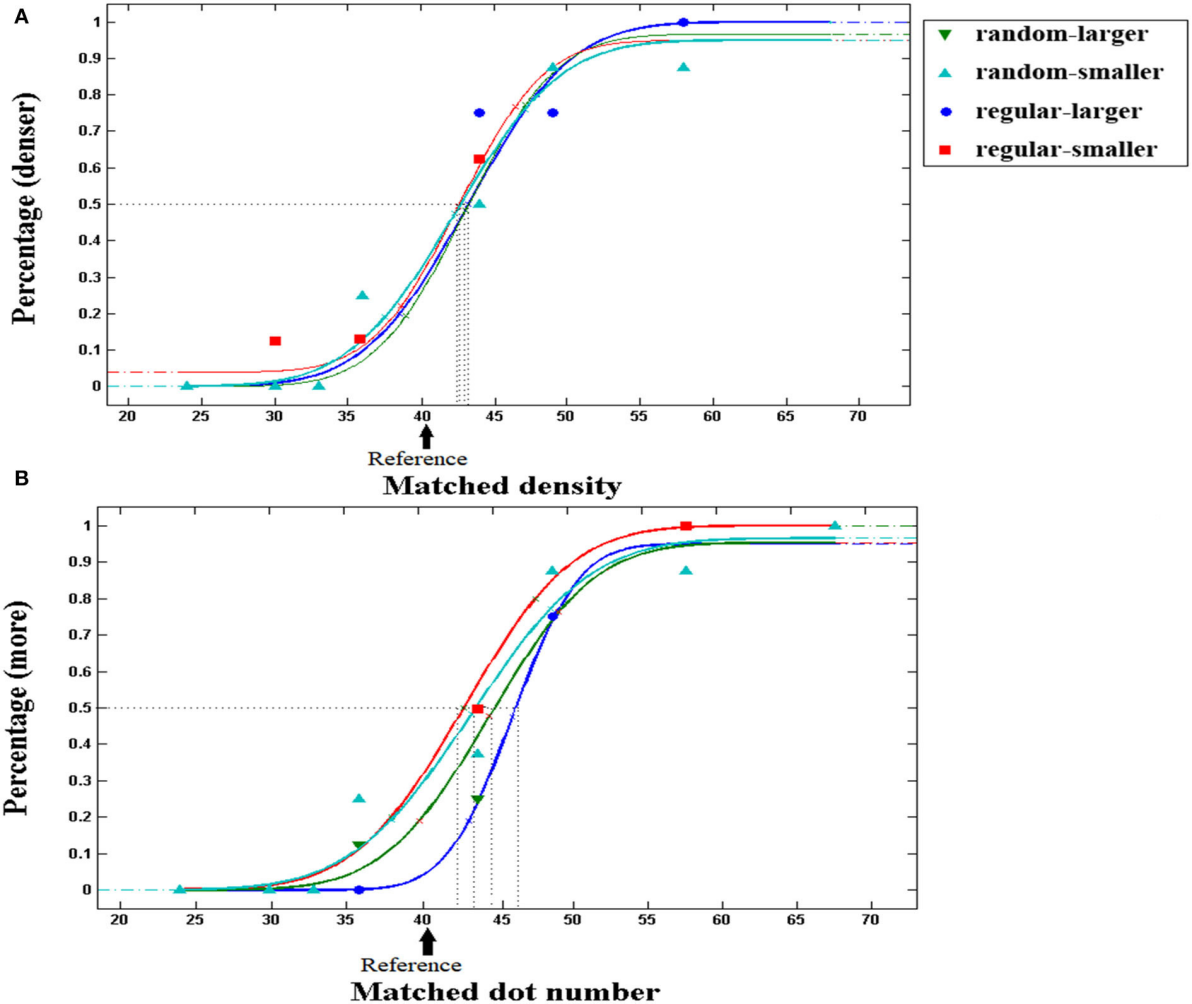
Typical psychometric functions in density and number tasks. The proportion of trials in which the test patch appeared to be denser/more numerous than the reference patch was plotted as a function of the density/number of the test dots, and the vertical dashed lines show the PSE. The arrow indicates the density/number of reference dots. The participants' typical responding curves are displayed to indicate the average PSE results. **(A)** Density task. **(B)** Number task. PSE shifts to the right in number comparison with regular patterns in perceived-larger patch (blue line), as compared with the smaller patch (red line). Note that certain fitting points were covered by others located in the neighborhood when new curves were generated.

Similarly to the size task, cumulative Gaussian models were fitted to the proportion of participants' responses (Figure 4A). The values of the test stimuli (abscissa) corresponding to the 50% points were calculated from the fitted curves (the parameter of α). These values are the PSE representing the density of test dots that appeared similar to that of the reference dots. The JND of the comparison can be indicated by the width of the fitted function (the parameter of β). The Weber fraction is defined as the division of JND by PSE (Anobile et al., 2014).

**Figure 5:**
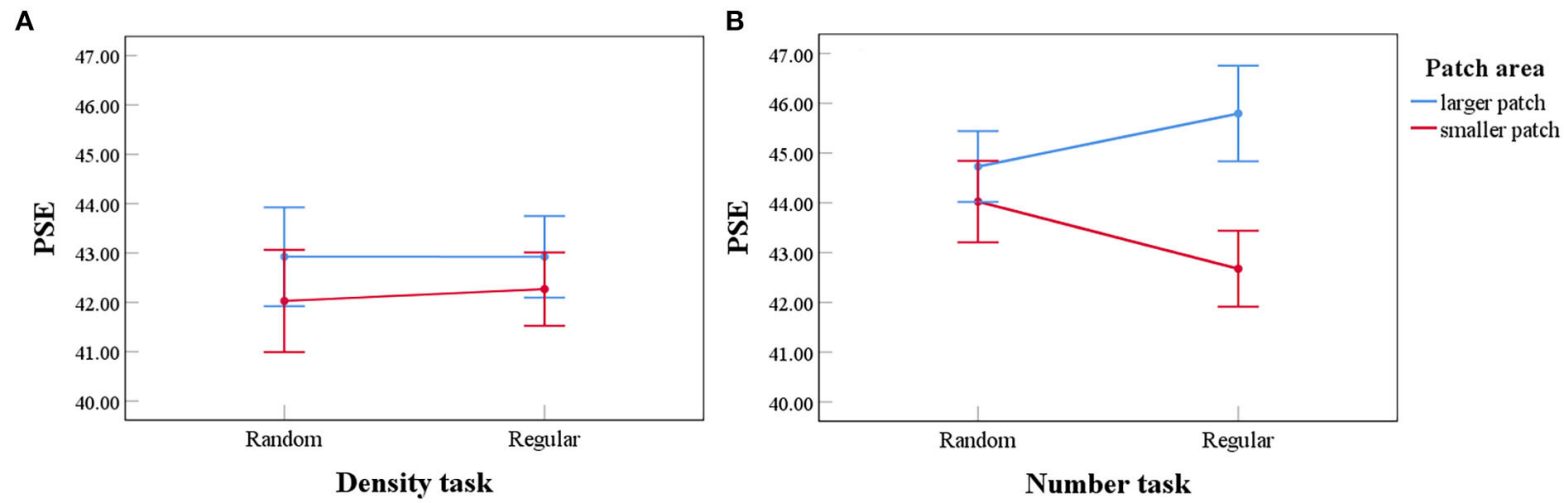
Results of a 2 (perceived patch area: larger/smaller) × 2 (distribution: random/regular) repeated measures ANOVA in density and number tasks. Error bars represent 1 standard error of the mean. For density tasks **(A)**, no main effect for the perceived patch area was found. No significant interaction was found, either. For number tasks **(B)**, there is a main effect for the perceived patch area. A significant interaction was found between perceived patch area and distribution. With regular patterns, a larger patch was perceived to be more numerous.

A 2 (perceived patch area: larger/smaller) × 2 (distribution: random/regular) repeated measures ANOVA was conducted with PSE (averaged between left and right locations) for comparing density as a dependent variable. The results are shown in Figure 5A. No significant main effect for perceived patch area was revealed, *F* (1, 33) = 2.236, *p* =0.144, $\eta_{p}^{2}$ = 0.063, *BF*_10_ = 0.385. No significant main effect was found for distribution, *F* (1, 33) = 0.020, *p* =0.888, $\eta_{p}^{2}$ = 0.001, *BF*_10_ = 0.183, and no significant interaction was revealed, either, *F* (1, 33) = 0.089, *p* =0.768, $\eta_{p}^{2}$ = 0.010, *BF*_10_ = 0.243. As expected, density comparison is not influenced by unmatched patch sizes.

### Number task

A 2 (perceived patch area: larger/smaller) × 2 (distribution: random/regular) repeated measures ANOVA was conducted with PSE for comparing numbers as a dependent variable (Figures 4B, 5B). Significant main effect for the perceived patch area was revealed, *F*(1, 33) = 10.205, *p* = 0.003, $\eta_{p}^{2}$ = 0.236, *BF*_10_ = 7.076. No significant main effect was found for distribution, *F*(1, 33) = 0.030, *p* =0.864, $\eta_{p}^{2}$ = 0.001, *BF*_10_ = 0.179. Significant interaction showed up, *F*(1, 33) = 5.127, *p* =0.030, $\eta_{p}^{2}$ = 0.134, *BF*_10_ = 1.033. Importantly, perceived area showed no effect on the PSE for comparing random patterns, *F*(1, 33) = 0.851, *p* =0.363, $\eta_{p}^{2}$ = 0.025, *BF*_10_ = 0.272. By contrast, it significantly affected the PSE for comparing regular patterns, *F*(1, 33) = 13.871, *p* = 0.001, $\eta_{p}^{2}$ = 0.296, *BF*_10_ = 42.321.

PSE for comparing regular patterns is biased according to the perceived area. When two patches contain an equal number of dots, the patch with a larger perceived area seems to be more numerous, even when the two patches appear equally dense, as suggested by the density task (Figure 5A). We propose that density and area analyses mediate the number comparison with regular patterns. When the two patches are equal in number, participants should perceive an equal density, and automatically infer that the larger patch contains more dots. By contrast, number comparison with random patterns is not biased by perceived area, which is consistent with our main hypothesis. It is in conflict with the proposal that the perception of numbers is based on the combination of density and area analysis. If this was the case, then with equivalent area disparity caused by the Ebbinghaus illusion, and identical testing series, similar overestimation in numerosity should occur. Contrary to this, the perception of number shows the signature of a direct encoding.

**Figure 6:**
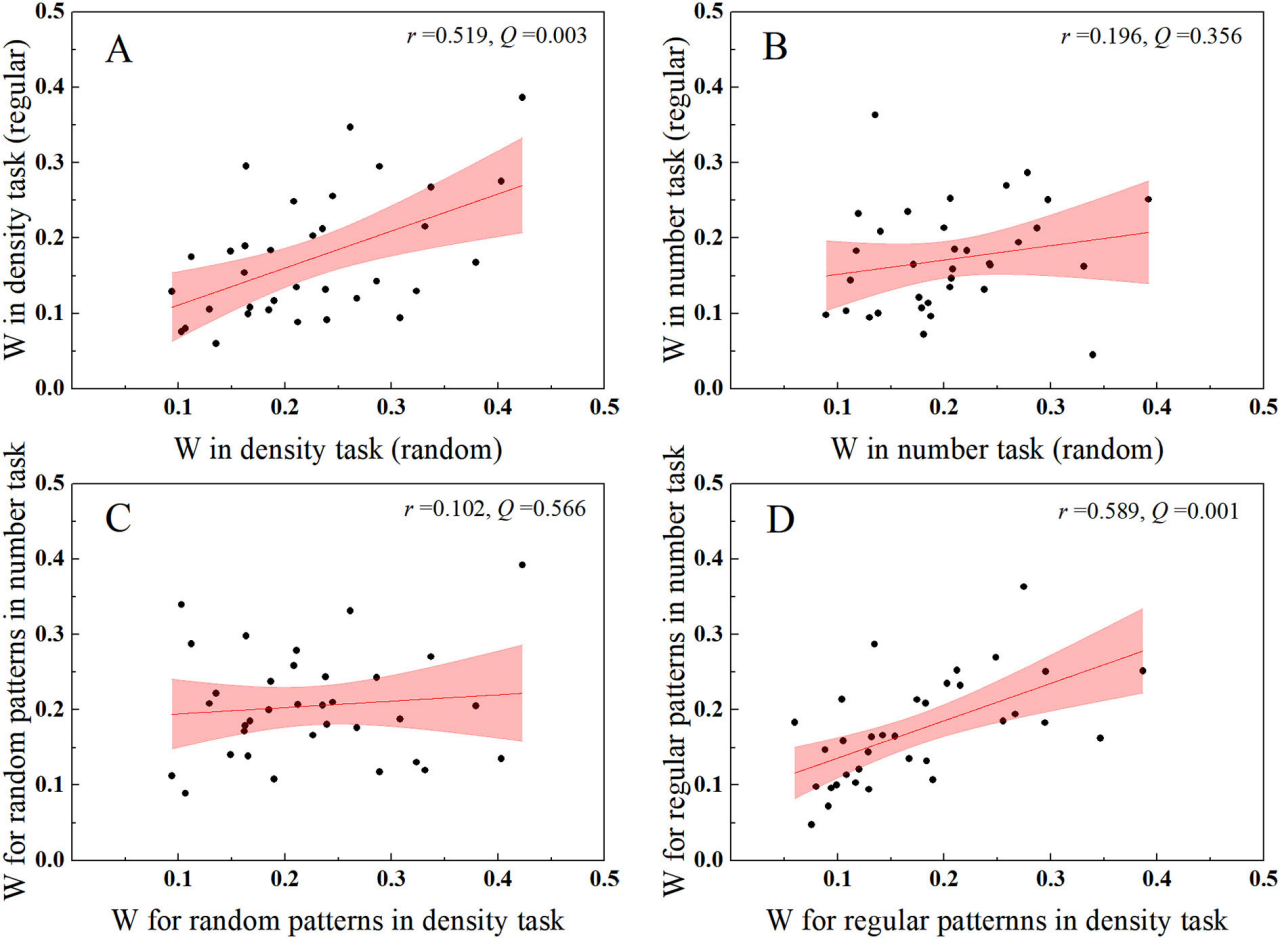
Scatters for individual Weber fractions between conditions either with the same task instruction or with the same distribution. **(A,B)** A significant correlation was found between patterns in the density task, whereas no correlation was found between patterns in the number task. **(C,D)** No significant correlation was found for Weber fractions of random patterns between tasks, whereas a significant correlation was found for Weber fractions of regular patterns between tasks. The pink area donates 95% CI.

To further support the idea that the number of regular dots is mediated by density processing in the number task, we calculated the correlation coefficients of individual Weber fractions between two test conditions either with the same instruction or with the same distribution (Figure 6). The Weber fraction estimates one's ability or precision for perceiving certain attributes. Individual Weber fractions for tasks based on a single mechanism should be correlated, whereas those for tasks based on independent mechanisms may not be correlated. In the number task, even with identical task instruction, no significant correlation was found for individual Weber fractions between random and regular patterns (Figure 6B), *r* = 0.196, *p* = 0.267, *Q* = 0.356, *BF*_10_ = 0.386, suggesting that distinct mechanisms took part in the enumeration of two patterns. On the contrary, in the density task, the Weber fractions of the two patterns were significantly correlated (Figure 6A), *r* = 0.519, *p* = 0.002, *Q* = 0.003, *BF*_10_ = 24.437, indicating that the density comparison of the two patterns was based on a single mechanism, i.e., the density mechanism. Taking a further look at the correlations between different tasks with identical distribution, the Weber fractions for comparing random dots were not correlated between tasks (Figure 6C), *r* = 0.102, *p* = 0.566, *Q* =0.566, *BF*_10_ = 0.250, demonstrating that distinct instructions succeeded in activating distinct mechanisms in the two tasks, whereas the Weber fractions were significantly correlated for regular patterns between tasks (Figure 6D), *r* = 0.589, *p* < 0.001, *Q* = 0.001, *BF*_10_ = 131.335, suggesting that the density mechanism was automatically activated by regular patterns, no matter the participants were asked to compare the number or density of the two patches.

We then analyzed the mean of Weber fractions. In density task, the mean was 0.225 (SD = 0.088) for random patterns and was 0.176 (SD = 0.083) for regular patterns. In the number task, the mean was 0.200 (SD = 0.072) for random patterns, and was 0.173 (SD = 0.070) for regular patterns. Paired *t*-test with correction of multiple comparisons showed a significant difference between two patterns in the density task, *t*(33) = 3.641, *p* < 0.001, *Q* =0.004, Cohen's *d* = 0.624, *BF*_10_ = 34.446, regular patterns are perceived with lower noise, even in the same density task. A marginal difference was revealed between the two patterns in the number task, which failed to reach the significant criterion after multiple comparison correction, *t*(33) = 2.153, *p* = 0.039, *Q* = 0.078, Cohen's *d* = 0.369, *BF*_10_ = 1.400. Weber fractions with random patterns showed no significant difference between density and number tasks, *t*(33) = 1.508, *p* = 0.298, *Q* = 0.397, Cohen's *d* = 0.181, *BF*_10_ = 0.307. No significant difference was found between the two tasks for regular patterns, either, *t*(33) = 0.045, *p* = 0.964, *Q* =0.964, Cohen's *d* = 0.008, *BF*_10_ = 0.184.

## Discussion

This study investigates the role of area and density in the judgement of numerosity. the Ebbinghaus illusion paradigm was adopted to induce disparity in the perceived area while the physical area was kept constant for the two patches to be compared, and participants were asked to compare the density or number of dots in two patches in different tasks. PSE, JND, and Weber fractions were computed from the psychometric functions fitted to the proportion of participants' responses. To this end, we found that for random patterns, the unmatched perceived area does not bias the PSE in density or number tasks. For regular patterns, inequality in the perceived area has no significant effect on PSE in the density task; however, in the number task, it leads to overestimation in the PSE for dots in the perceived-larger patch.

Zimmermann and Fink (2016) employed size adaptation to manipulate the apparent size of numerosity patches while maintaining the physical patch area. Density perception was found to be invariant to the varying area, and changes in the apparent area had a much weaker effect on numerosity perception for low numerosities (4–25) within the numerosity regime (Anobile et al., 2014, 2015) than for high numerosities (50–100). It was suggested that low numerosities are sensed directly, whereas high numerosities are inferred on the basis of visual cues such as size and density (Zimmermann and Fink, 2016). Our results are largely consistent with those of the previous study except that there is no measurable PSE bias in number tasks for random patterns with low numerosities (40 dots). Area distortion was induced using different paradigms in the two studies, which may explain the inconsistency. The simultaneous adaptation in area and numerosity may be due to overlapping representations in the intraparietal cortex (Pinel et al., 2004; Zimmermann and Fink, 2016).

It is suggested that both the numerosity and density mechanisms can be activated in number tasks and that dots can disrupt the numerosity mechanism when they are too dense to be separated (Anobile et al., 2014, 2015). Our previous study pointed out that high regularity, as well as high density, can disrupt approximate numerosity processing by affecting individuation (He et al., 2009; Liu et al., 2017, 2018). A series of studies revealed behavioral and ERP dissociation in number processing between random and regular patterns, consistent with the dissociation between numerosity and density mechanisms (Liu et al., 2017, 2018, 2022), suggesting that regular distribution can trigger the density mechanism in number tasks. In this study, individual Weber fraction analysis provides further evidence. There is no correlation between the Weber fractions of comparison with regular and random patterns in the number task, but the former is significantly correlated with the Weber fractions of both patterns in the density task. Despite participants being asked to decide which patch is more numerous, the regular patterns activate the mechanism of density rather than numerosity. Regular dots presented in perceived-larger patches induce no bias in density comparison, whereas they are regarded as more numerous. These results further support that by combining the results of perceiving density and area, the number can be inferred when the approximate number processing is disrupted by dot distribution.

For random patterns, no bias was found, either in number or density tasks. Researchers have previously found that dots in larger patches are perceived to be both denser and more numerous (Dakin et al., 2011; but see the studies of Allik et al., 1991; Bell et al., 2015). The present results contradict this earlier study.

First, we examined the explanation that some participants may realize that the patches were actually equal in size, so they might compare the density of the random patterns regardless of whether they were asked to compare density or number. This inference suggests that the absence of bias in numerosity comparisons with random dots is due to a cognitive strategy. The theory cannot explain why a similar strategy was not taken in the regular group, in which significant overestimation of numerosity was observed in the larger patch. The lack of correlation for Weber fractions between number and density tasks with random patterns also contradicts this theory.

Second, we analyzed the hypothesis that the Ebbinghaus illusion may occur beyond the processing of numerosity and density. According to a previous study, a mismatch in numerosity caused by the connectedness illusion (He et al., 2009) does not result in a bias on the perceived duration, while a mismatch in physical numerosity does affect the perceived duration, suggesting that the influence of numerosity on duration occurs before the connectedness illusion (Togoli et al., 2021). The Ebbinghaus illusion, however, is encoded and computed to a large extent along the ventral visual stream, including the early visual cortex like V1 and V2/V3, and the posterior temporal cortex (Chen et al., 2021). Thus, the absence of effect cannot be explained as the Ebbinghaus illusion emerges after the processing of numerosity and density.

Third, the current results cannot predict how systematically changing area would affect numerosity and density perception. The Ebbinghaus illusion induces a size difference of about 24% between the two patches, similar to a previous study based on a similar rationale (Zimmermann and Fink, 2016), but it is smaller than the physical size difference adopted in previous studies (Dakin et al., 2011; Bell et al., 2015). It is possible that larger area differences may induce measurable PSE bias in numerosity (Dakin et al., 2011; but see the studies of Allik et al., 1991; Bell et al., 2015) and density perception (Dakin et al., 2011; Bell et al., 2015).

Nevertheless, this study clearly supports the notion that numerosity with random patterns is sensed directly via neurons that are tuned for specific numerosities (Viswanathan and Nieder, 2013; Zimmermann and Fink, 2016). The results that neither number nor density comparison is biased in favor of the perceived area rule out the possibility that numerosity is inferred by combining area and density information in the context of random patterns. It is worth noting that the size disparity induced by the Ebbinghaus illusion is similar for the two patterns, and the testing series are also identical. If numerosity was inferred by combining density and area, then due to no bias in perceiving density and a bias in perceiving area, significant overestimation of numerosity should be observed for the larger patch, similar to what occurs in the regular group, even with an area disparity of 24%. However, the results suggest that randomly distributed dots can be sensed directly based on their numerosity. In addition, the perception of numerosity and density should operate independently, as the Weber fractions for random patterns are not correlated between tasks.

## Conclusion

For random patterns, area inequality induced by the Ebbinghaus illusion does not affect the perception of number and density. Numerosity and density mechanisms operate independently. For regular patterns, density perception is unaffected by area inequality for regular patterns, whereas numerosity is significantly overestimated in the perceived larger patch. Weber fractions for regular patterns are correlated between number and density tasks. Numerosity is sensed directly with random patterns, whereas when the approximate numerosity mechanism is disrupted by dot distribution, numerosity is inferred on the basis of density and area.

## Data availability statement

The original contributions presented in the study are included in the article/supplementary material, further inquiries can be directed to the corresponding author.

## Ethics statement

The studies involving human participants were reviewed and approved by Ethics Committee of the Yunnan Minzu University. The patients/participants provided their written informed consent to participate in this study. Written informed consent was obtained from the individual(s) for the publication of any potentially identifiable images or data included in this article.

## Author contributions

WL and ZZ developed the study concept and contributed to the study design. CW and SD performed the testing and data collection and provided critical revisions. WL performed the data analysis, interpretation, and drafted the manuscript. CW and XZ drew the figures. All authors contributed to manuscript revision, read, and approved the submitted version.

## Funding

This study was supported by the Funds of the National Natural Science Foundation of China (Grant No. 32060192) and the Funds of the Philosophy and Social Science Foundation of Yunnan Province (Project of Education, Grant No. AC20014).

## Conflict of interest

The authors declare that the research was conducted in the absence of any commercial or financial relationships that could be construed as a potential conflict of interest.

## Publisher's note

All claims expressed in this article are solely those of the authors and do not necessarily represent those of their affiliated organizations, or those of the publisher, the editors and the reviewers. Any product that may be evaluated in this article, or claim that may be made by its manufacturer, is not guaranteed or endorsed by the publisher.

## References

[B1] Allik J. Tuulmets T. Vos P. G. (1991). Size invariance in visual number discrimination. Psycho Res. 53, 290–295. 10.1007/BF00920482 1792300

[B2] Anobile G. Cicchini G. M. Burr D. C. (2014). Separate mechanisms for perception of numerosity and density. Psychol. Sci. 25, 265–270. 10.1177/0956797613501520 24270462

[B3] Anobile G. Turi M. Cicchini G. M. Burr D. C. (2015). Mechanisms for perception of numerosity or texture-density are governed by crowding-like effects. J. Vis. 15, 1–12. 10.1167/15.5.4 26067522 PMC4909146

[B4] Bell J. Manson A. Edwards M. Meso A. (2015). Numerosity and density judgments: Biases for area but not for volume. J. Vis. 15, 18. 10.1167/15.2.18 25761336

[B5] Beran M. J. and Parrish, A. E.. (2016). “Going for more: discrete and continuous quantity judgments by nonhuman animals,” in Continuous Issues in Numerical Cognition, ed A. Henik, 175–192. 10.1016/B978-0-12-801637-4.00008-1

[B6] Burr D. C. (2017). Evidence for a number sense. Behav. Brain Sci. 18, e167. 10.1017/S0140525X16002077 29342625

[B7] Burr D. C. Ross J. (2008). A visual sense of number. Curr. Biol. 18, 425–428. 10.1016/j.cub.2008.02.052 18342507

[B8] Chen L. Xu Q. Shen L. Yuan T. Wang Y. Zhou W.. (2021). Distinct contributions of genes and environment to visual size illusion and the underlying neural mechanism. Cereb. Cortex 32, 1–10. 10.1093/cercor/bhab262 34379728 PMC8889949

[B9] Cicchini G. M. Anobile G. Burr D. C. (2016). Spontaneous perception of numerosity in humans. Nat. Commun. 7, 12536. 10.1038/ncomms12536 27555562 PMC4999503

[B10] Cicchini G. M. Anobile G. Burr D. C. (2019). Spontaneous representation of numerosity in typical and dyscalculic development. Cortex 114, 151–163. 10.1016/j.cortex.2018.11.019 30683323

[B11] Dakin S. C. Tibber M. S. Greenwood J. A. Kingdom F. A. Morgan M. J. (2011). A common visual metric for approximate number and density. Proc. Nat. Acad. Sci. 108, 19552–19557. 10.1073/pnas.1113195108 22106276 PMC3241748

[B12] Dehaene S. (2002). Précis of The Number Sense. Mind Lang. 16, 16–36. 10.1111/1468-0017.00154

[B13] Dehaene S. Changeux J. P. (1993). Development of elementary numerical abilities: a neuronal model. J. Cogn. Neurosci. 5, 390–407. 10.1162/jocn.1993.5.4.390 23964915

[B14] Fornaciai M. Park J. (2017). Distinct neural signatures for very small and very large numerosities. Front. Hum. Neurosci. 11, 21. 10.3389/fnhum.2017.00021 28197086 PMC5282473

[B15] Gebuis T. Reynvoet B. (2013). The neural mechanisms underlying passive and active processing of numerosity. NeuroImagine 70, 301–307. 10.1016/j.neuroimage.2012.12.048 23282277

[B16] He L. Zhang J. Zhou T. Chen L. (2009). Connectedness affects dot numerosity judgment: Implications for configural processing. Psychon. Bull. Rev. 16, 509–517. 10.3758/PBR.16.3.509 19451377

[B17] Leibovich T. Katzin N. Harel M. Henik A. (2017). From “sense of number” to “sense of magnitude”: The role of continuous magnitudes in numerical cognition. Behav. Brain Sci. 40, e164. 10.1017/S0140525X16000960 27530053

[B18] Liu W. Zhang Z. J. Zhao Y. J. Li B. C. Wang M. (2017). Distinct mechanisms in the numerosity processing of random and regular dots. Acta Psychol. 3, 17–30. 10.1016/j.actpsy.2017.01.006 28131034

[B19] Liu W. Zhao Y. Wang C. Wang L. Fu Y. Zhang Z. (2022). Distinct mechanisms in number comparison of random and regular dots: an ERP study. Front. Behav. Neurosci. 15, 791289. 10.3389/fnbeh.2021.791289 35095437 PMC8789750

[B20] Liu W. Zhao Y. J. Wang M. Zhang Z. J. (2018). Regular distribution inhibits generic numerosity processing, Front. Psychol. 9, 2080. 10.3389/fpsyg.2018.02080 30429812 PMC6220036

[B21] Liu W. Zheng P. Huang S. Cicchini G. M. (2020). Subitizing, unlike estimation, does not process sets in parallel. Sci Rep. 10, 15689. 10.1038/s41598-020-72860-4 32973306 PMC7518424

[B22] Pinel P. Piazza M. Le Bihan D. Dehaene S. (2004). Distributed and overlapping cerebral representations of number, size, and luminance during comparative judgments. Neuron. 41, 983–993. 10.1016/S0896-6273(04)00107-2 15046729

[B23] Takao S. Watanabe K. Cavanagh P. (2021). Dynamic presentation boosts the Ebbinghaus illusion but reduces the Müller-Lyer and orientation contrast illusions. J. Vis. 21, 4. 10.1167/jov.21.6.4 34110368 PMC8196426

[B24] Titchener E. B. (1905). Experimental Psychology: A Manual of Laboratory Practice. New York: Macmillan.

[B25] Togoli I. Fornaciai M. Bueti D. (2021). The specious interaction of time and numerosity perception. Proc. R. Soc. B. 288, 20211577. 10.1098/rspb.2021.1577 34547911 PMC8456131

[B26] Viswanathan P. Nieder A. (2013). Neuronal correlates of a visual “sense of number” in primate parietal and prefrontal cortices. Proc. Natl. Acad. Sci. 110, 11187–11192. 10.1073/pnas.1308141110 23776242 PMC3704030

[B27] Yousif S. R. Keil F. C. (2020). Area, not number, dominates estimates of visual quantities. Sci. Rep. 10, 1–13. 10.1038/s41598-020-68593-z 32770093 PMC7414215

[B28] Zimmermann E. Fink G. R. (2016). Numerosity perception after size adaptation. Sci. Rep. 6, 32810. 10.1038/srep32810 27650296 PMC5030660

